# Delay in Cutaneous Squamous Cell Carcinoma Diagnosis Due to Interrupted Services Is Associated with Worse Prognoses and Modified Surgical Approaches

**DOI:** 10.3390/cancers16081469

**Published:** 2024-04-11

**Authors:** Filippo Taccioli, Claudio Gio Francesco Blessent, Alessia Paganelli, Francesca Fagioli, Johanna Mary Chester, Shaniko Kaleci, Matteo Costantini, Barbara Ferrari, Chiara Fiorentini, Giorgio De Santis, Cristina Magnoni

**Affiliations:** 1 Department of Plastic Reconstructive and Aesthetic Surgery, Azienda Ospedaliero-Universitaria di Modena, University of Modena and Reggio Emilia, 41125 Modena, Italy; desantis.giorgio@aou.mo.it; 2 PhD Course in Clinical and Experimental Medicine, Department of Biomedical, Metabolic and Neural Sciences, University of Modena and Reggio Emilia, 41125 Modena, Italy; alessia.paganelli@gmail.com; 3 Health Directorate, Azienda Ospedaliero-Universitaria di Modena, University of Modena and Reggio Emilia, 41125 Modena, Italy; fagioli.francesca@aou.mo.it; 4 Surgical, Medical, Dentistry and Morphological Sciences, University of Modena and Reggio Emilia, 41125 Modena, Italy; johanna.chester@unimore.it (J.M.C.); shaniko.kaleci@unimore.it (S.K.); 5 Institute of Pathology, University of Modena and Reggio Emilia, 41125 Modena, Italy; matteo.costantini@aou.mo.it; 6 Department of Dermatology, University of Modena and Reggio Emilia, 41125 Modena, Italy; ferrari.barbara1@gmail.com (B.F.); fiorentini.chiara@aou.mo.it (C.F.); cristina.magnoni@unimore.it (C.M.)

**Keywords:** cutaneous squamous cell carcinomas, COVID-19, delayed diagnosis, skin cancer, surgery

## Abstract

**Simple Summary:**

The timely diagnosis and surgical treatment of skin squamous cell carcinomas is crucial for patient outcomes. However, interruptions to health care services, such as a pandemic, natural disaster, or war, can affect patients’ willingness to seek medical assistance. We investigated the impact of the delayed diagnosis of squamous cell carcinomas of the skin by comparing patient and carcinoma characteristics before (2018–2019) and after (2021–2022) the second wave of the COVID-19 pandemic. We found that patients were less likely to seek medical assistance, and when they did, carcinomas were more advanced. To curb the spread of the virus, types of surgery changed.

**Abstract:**

Background: The delayed diagnosis of skin tumors is associated with a worsened prognosis. The impact of the interruption of clinical and surgical health services during the COVID-19 pandemic lockdowns has been documented among many pathologies. The impact of delayed diagnoses on patients with cutaneous squamous cell carcinomas (cSCCs) is poorly defined. Objective: To compare patient and lesion characteristics and the surgical management of excised cSCCs prior to the pandemic shutdown of services (2018–2019) with the phase following the pandemic’s second wave (2021–2022). Methods: An observational, single-center, cross-sectional study of 416 surgically excised cSCCs over the course of two years was performed. Only patients with histologically confirmed cSCC were enrolled. Data collection included patient demographics and lesion characteristics, time to surgery, surgical approach, and histological data. Results: More cSCC lesions were excised prior to the interruption of services (*n* = 312 vs. *n* = 186). Lesions were significantly larger (1.7 ± 1.2 vs. 2.1 ± 1.5 cm; *p* = 0.006) and more invasive (52% vs. 89%; *p* < 0.001), in the period 2021–2022. Surgical reconstructive techniques were significantly different (*p* = 0.001). Metastatic involvement was confirmed in three subjects (one in 2018–2019 and two in 2021–2022). There were no significant differences in the time to surgery or patient characteristics. Multivariable regression analysis identified a 4.7-times higher risk of tumor invasion (OR 4.69, 95%CI 2.55–8.16, *p* < 0.001), a two-times higher chance of dermo-epidermal grafts (OR 2.06, 95%CI 1.09–3.88, *p* = 0.025), and a 3.2-times higher risk of positive surgical margins (OR 3.21, 95%CI 1.44–7.17, *p* = 0.004). Conclusions: Diagnostic delays of cutaneous SCCs associated with reduced patient access to clinical and diagnostic services are associated with a 4.7-times increased risk of more severe invasion, a three-times increased risk of positive surgical margins, and a significant impact on surgical management, compared to the pre-pandemic period. Comparable patient cohort characteristics and time to surgery remained unchanged.

## 1. Introduction

To limit the spread of the COVID-19 virus and the subsequent pressure on health care services, authorities worldwide reduced or limited non-emergent medical services and procedures, including cancer screening and surgery [1,2,3]. Reduced access to standard screening and diagnostics pathways due to the COVID-19 pandemic created both novel challenges and downstream effects from the reallocation of resources away from other patient groups requiring time-critical access to health care services [4,5]. Furthermore, the number of people seeking health care was greatly impacted by the perceived risk of exposure to the virus. 

Restrictions also affected the strategies of prioritization to determine which patients should undergo surgery, since the resource scarcity (anesthetists, nurses, personal protective equipment) resulted in few patients treated [6]. Some authors have reported reduced oncological surgical interventions during the pandemic period [7] as expected. However, the effects of interrupted services for patients with cancer is not immediate, with premature death expected to be registered even years after the pandemic due to missed or postponed diagnosis and/or treatment. However, due to variations in both clinical practice and the types of presenting tumors, attempts at modeling the impact of delayed oncological services and reduced cancer patient access on morbidity and mortality have been challenging [4]. Furthermore, recommendations on the surgical intervention to offer tended to prefer those with a reduced hospital stay (limiting COVID-19 exposure) or same-day procedures with post-operative home rehabilitation [8].

Studies have been performed to measure the impact of the reduced capacities of diagnostic and surgical services in distinct cancer types, such as lung, colorectal, breast, and esophageal cancers [4].

Cutaneous squamous cell carcinoma (cSCC) is a common skin cancer characterized by the malignant proliferation of the keratinizing cells of the epidermis or its appendages. It is the second most common form of non-melanoma skin cancer (NMSC) [9] and it is estimated that cSCC represents about 20–25% of all skin neoplasms in Italy [10]. Invasive cSCC is known to impact morbidity, mortality, and patients’ quality of life [11,12]. Unlike other types of non-melanoma skin cancer (NMSC), cSCC carries the risk of loco-regional and/or distant spreading [13]. Since delayed diagnosis can lead to metastases, early diagnosis has been deemed essential [14].

We aim to provide evidence of the impact of restrictions to diagnostic and surgical services on cSCCs, at a single center in one of the worst COVID-19 pandemic-affected Italian regions, comparing excised cSCC lesions prior to (2018–2019) and following (2021–2022) the pandemic’s second wave. We also aim to identify any significant changes in terms of patients’ characteristics, surgical interventions, lesions’ comparative size, level of invasion and positive surgical margins, and nodal metastasis. We assume that the period of restrictions unavoidably contributed to a worsening of the cSCCs considered and a modification of surgical approach.

## 2. Materials and Methods

### 2.1. Patient Selection and Data Collection

An observational, single-center, cross-sectional study was performed on consecutive primary cSCCs treated with postoperative margin assessment and wide local excision, and with postoperative margin assessment and confirmed histopathological diagnosis, identified over two 12-month periods: from June 2019 to June 2020 and from June 2021 to June 2022. Patients meeting these criteria were identified from an institutional pathology database, treated at the Dermatologic Surgery Department of Modena University Hospital. Patients with incisional biopsies or non-cutaneous SCCs were excluded. 

Patient data and tumor characteristics were retrospectively collected from medical records and pathological referrals and were stored in a dedicated Microsoft Excel database (Microsoft 2018). Data retrieved included patient sex and age, number of lesions per patient, tumor size, degree of differentiation, invasion, microinvasion, depth of invasion, perineural involvement (PNI), lymphatic/vascular involvement (LVI), time from diagnosis to surgery, surgical technique, hospitalization, surgical margins, and nodal metastases. When PNI, LVI, surgical margins, and metastasis were not stated in pathological reports, patients were assumed to be negative. 

### 2.2. Surgical Approach

The surgical approach was selected for each patient based on the lesion’s size and anatomical position, the patient’s age and skin quality, the patient’s ability to access post-operative care, and the length of the hospital stay. Depending on the site of the cancer and its size, many techniques are available to successfully reconstruct the surgical defects after tumor excision. 

Oncological recommendations for surgical adequacy specify that 5 mm of neoplasm-free skin should be incorporated into the resected specimen [15]. This has proven to break down the rate of local recurrences [16]. The “reconstructive ladder” [17] surgical principle states that the selection of reconstruction should start from the simplest techniques, such as secondary and primary intention, before pursuing more complex (and sometimes expensive) techniques, such as cutaneous flaps, dermo-epidermal grafts, and dermal matrix substitutes [18]. Interventions were carried out according to the guidelines provided by the Italian Association of Medical Oncology [10] and the National Comprehensive Cancer Network [19]. Clinical approaches during the first wave of the pandemic (March 2020–June 2020) were modified at our center, with surgical priority given to invasive melanoma, histologically confirmed infiltrating high-risk SCC, and rapidly growing nodular lesions. Excisions for undiagnosed flat non-melanoma skin cancer (NMSC), and wide excisions for in situ sCC or BCC were postponed, as has been previously described [20]. Following the second wave of the pandemic, the selection of the surgical approach was heavily influenced by the required length of hospital stay, preferring easier, quicker, and safer surgical techniques such as dermo-epidermal graft and ADM, especially on older and frail patients, in order to minimize the exposure to COVID-19 during patients’ hospitalization.

### 2.3. Statistical Analysis

Statistical analysis was performed using STATA^®^ software version 17 (StataCorp. 2021. Stata Statistical Software: Release 17. College Station, TX, USA: StataCorp LLC.). Descriptive statistics were presented for the baseline demographic clinical characteristics for the entire group, as well as for the groups of patients 2019–2020 and 2021–2022. Continuous variables were presented as the number of patients (*n*), mean, standard deviation (SD), minimum (min), and maximum (max) and compared between subgroups using an unpaired Student’s *t* test. Categorical variables were presented as frequency (*n*, percentage [%]) and compared using Pearson’s chi-squared test. Univariate and multivariate logistic regression models were carried out using a stepwise selection method to identify the prognostic factors between patient groups. In the first step, the intercept-only model was fitted with individual score statistics for potential variables. A significance level of *p* < 0.05 was used to allow a variable into the model. The Hosmer–Lemeshow test was used to evaluate “goodness of fit” in the selection model. Data from the univariate and multivariate logistic regression analyses were expressed as odds ratio (OR) and 95% confidence interval (CI). The Bonferroni correction was conducted on multiple statistical comparisons. The results of the statistical tests were then interpreted using the corrected alpha level to determine statistical significance. *p* < 0.05 was considered statistically significant.

## 3. Results

A total of 498 cSCC lesions (416 patients) met the inclusion criteria and were included in the present study. For a complete description of the included patients and lesions, see Table 1. The comparative number of excised cSCCs reduced by 40% in the period following the second wave of the pandemic (*n* = 310 [265 patients] vs. *n* = 186 [161 patients]). 

Excised tumors were significantly larger, more often undifferentiated, and more often invasive in the period following the post-pandemic’s second wave compared to the pre-pandemic period (*p* = 0.006, *p* = 0.007, *p* < 0.001). There were also significantly fewer in situ and microinvasive cSCCs excised in the post-pandemic second wave period (*p* < 0.001, *p* = 0.010). There were no differences observed in the average time to surgery but the surgical techniques significantly changed in favor of approaches requiring a shorter hospital stay in the post-pandemic second wave period (*p* = 0.001). The frequency of positive surgical margins significantly increased in the second period (0.019). Lymphatic metastases were rarely observed: only one patient in the pre-pandemic period and two in the post-pandemic second wave period. However, no significant difference in terms of nodal or distant metastases was detected between the two groups.

Univariate analysis confirmed the statistical differences outlined in Table 1. Multivariable regression analysis identified a 4.7-times increased risk of tumor invasion, a 7–9-times increased risk of reticular, dermis, and hypodermis invasion, a two-times augmented chance of surgical management with dermo-epidermal grafts, and a 3.2-times higher risk of positive surgical margins, compared to the pre-pandemic period (see Table 2).

## 4. Discussion

Our study provides evidence that restrictions to diagnostic and surgical services significantly impact the clinical presentation of cSCC and their management. Among our cohort, lesions were more invasive, managed differently, and were more likely to have positive surgical margins following the second wave of the COVID-19 pandemic, despite similar baseline risk factors and time to surgery. Fewer patients presented themselves for lesion assessment following the second wave of the pandemic, with significantly fewer in situ cSCCs excised. 

Initial modeling of the impact of mitigating viral spread and protecting hospital staff and resources through restrictions foresaw significant changes in cancer severity presentation, with consequent increased morbidity and mortality. Estimates varied according to cancer type [21,22,23]. However, modeling was reported to be difficult, given the heterogeneity of recommendations and responses worldwide [4]. Further, the modeling and initial results of impact have focused on the main tumor types, including lung, breast, colorectal, and esophageal. The impacts of the pandemic on minor tumor types also need to be assessed and reported.

During the interruption to services, our dermatological oncological department prioritized melanoma and infiltrating cSCC for timely excision, as previously described [20]. Our results prove that there was a 40% reduction in cSCC diagnoses at our center, suggesting that patients’ health-seeking behavior changed [24]. McClean et al. also reported an overall reduction in SCC diagnoses of 28% in 2020 in their single-center study [25], confirming the phenomenon of reduced patient presentation due to the perceived risk of COVID-19 disease exposure.

Diagnostic delay was expected to translate into clinical observations of more advanced lesions, requiring more invasive surgery, with an increased chance of metastasis [26]. Our efforts of data retrieval confirm that the reduced cohort of presenting patients were diagnosed with lesions at a later stage of disease; in addition, lesion diameter, tumor grade, and local invasion of excised lesions were significantly higher with more local invasion following the second wave of the pandemic. Diaz-Cavillo et al. reported similar findings of an increased rate of high-risk cSCC in their Spanish-based study [27].

The risk of metastasis or death is higher for patients with tumors with an increased depth of invasion, a large tumor dimension (≥2 cm), poorly differentiated disease, perineural invasion of larger nerves (≥0.1 mm in dimension), and immunosuppression therapies [28,29].

Treatment delay for patients with cSCC is associated with some interval of progression in the size of the tumor [30]. However, as stated by Baumann et al., to our knowledge there are no data demonstrating a statistically significant association between treatment delay and an increased risk of disease-specific mortality [31].

The surgical approach to cSCC excision was altered due to pandemic restrictions and the necessity to reduce the pandemic’s viral spread [31]. In suitable patients, interventions performed in day surgery were preferred. The use of dermal-epidermal grafts doubled in frequency among patients treated in the second study period. However, of note, the frequency of positive surgical margins also significantly increased; previous studies have proven that increased lesion diameter and invasive subtypes are significant risk factors for positive margins [32,33].

More advanced lesions are associated with a higher risk of metastasis [34]. The occurrence of metastasis associated with cSCC diagnoses is rare and the current study is underpowered for any observation of significant differences. However, a larger single-center, cross-sectional study (2019 vs. 2020) performed in the United Kingdom reported a significant increase in nodal and metastatic cSCC tumor stages following the initial wave of the COVID-19 pandemic [25]. This trend is concerning, as metastatic involvement in cSCC impacts both patient prognosis and the complexity of the patient pathway, requiring more aggressive, complex, and expensive therapies, which often lead to patient death (>70%) [35]. 

The implications of our research extend beyond the clinical management of non-melanoma skin cancer during and following a pandemic. Our research should contribute to the essential creation of resilient health care systems for any unanticipated environmental, social, or political challenge, in a resource-limited setting [36]. The management of altered patient accessibility to standard pathways, including effective communication with the public about the risks of delaying cancer assessment, needs to be woven into a future health care structure [7]. 

Our study presents some limitations, and this small cohort study should be interpreted with caution. Firstly, the research was conducted in a geographical region involved in the initial, rapid diffusion of the COVID-19 viral spread. Therefore, the impact on patients’ hesitation to access health care services may not be representative of the general Italian population. Our retrospective data analysis did not include the identification of the exact anatomical location, Breslow thickness, lifestyle habits, and other possible extrinsic factors. Follow-up data were not collected and analyzed and therefore, any impact on morbidity and mortality cannot be made; this limitation could be the foundation for a 5-year follow-up study in which we will evaluate the impact of these strategies we adopted on the long-term prognosis of our patients.

Further, there are limited studies available for effective comparisons of our results. 

## 5. Conclusions

Our study confirms a significant impact on the presentation of cSCCs following the second wave of the COVID-19 pandemic, with a 4.7-times increased likelihood of local invasion, causing significant changes in surgical management and a three-times increased risk of positive surgical margins, compared to the pre-pandemic period. Comparable patient cohort characteristics and time to surgery remained unchanged. 

## Figures and Tables

**Table 1:** Patient and lesion characteristics, and surgical data for the patient cohort and for the groups of patients treated in the pre-pandemic period (2019–2020) and the post-second wave period (2021–2022).

Patient Characteristics	Total (*n* = 416)	2019–2020 (*n* = 260, 62.5%)	2021–2022 (*n* = 156, 37.5%)	
Female	96 (23.1)	55 (21.1)	41 (26.3)	0.229
Age, mean yrs ± SD (range)	81.3 ± 10.2 (33–101)	80.7 ± 10.0 (40–101)	82.3 ± 10.5 (33–101)	0.126
Number of lesion(s)/patient, *n* (%)				
1	357 (85.8)	221 (85.0)	136 (87.2)	0.537
≥2	59 (14.2)	32 (12.3)	17 (10.9)	
LESION CHARACTERISTICS	Total (*n* = 498)	2019–2020 (*n* = 312, 62.7%)	2021–2022 (*n* = 186, 37.3%)	
Lesion diameter *n*, mean ± SD (range)	483, 1.8 ± 1.4 (0.1–12)	308, 1.7 ± 1.2 (0.1–9.5)	175, 2.1 ± 1.5 (0.5–12)	0.006
Degree of differentiation				
Well/moderately differentiated	404 (81.1)	264 (84.6)	140 (75.3)	0.007
Poorly differentiated/undifferentiated	81 (16.3)	40 (12.8)	41 (22.0)	
Missing	13 (2.6)	8 (2.6)	5 (2.7)	
Tumor invasion				
In situ	166 (33.3)	146 (46.8)	18 (9.7)	<0.001
Invasive	320 (64.3)	163 (52.2)	165 (88.7)	
Microinvasive	41 (12.8)	33 (20.2)	8 (4.8)	0.010
Missing	12 (2.4)	3 (1)	3 (1.6)	
Depth of invasion				
Papillary dermis	47 (9.4)	25 (8.0)	22 (11.8)	<0.001
Reticular dermis	123 (24.7)	50 (16.0)	73 (39.2)	
Dermis (unspecified)	81 (16.3)	37 (11.8)	44 (23.7)	
Hypodermis	27 (5.4)	21 (6.7)	6 (3.2)	
Muscle tissue	13 (2.6)	13 (4.2)	0 (0.0)	
Missing	76 (15.3)	51 (16.3)	25 (13.4)	
LVI and PNI				
LVI	2 (0.4)	2 (0.6)	0 (0.0)	0.162
PNI	34 (6.8)	17 (5.4)	17 (9.1)	
Nodal metastases	3 (0.6)	1 (0.3)	2 (1.1)	0.292
SURGICAL DATA				
Time to surgery, *n*, mean months ± SD (range)	496, 1.8 ± 1.8 (0.3–15.6)	311, 1.9 ± 1.9 (0.5–15.6)	185, 1.6 ± 1.7 (0.3–15.0)	0.152
Surgical technique				
Direct closure	243 (48.8)	166 (53.2)	767 (41.4)	0.001
Dermo-epidermal graft	101 (20.3)	46 (14.7)	55 (29.6)	
Dermal matrix	63 (12.6)	38 (12.2)	25 (13.4)	
Flap	91 (18.3)	62 (19.9)	29 (15.6)	
Hospitalization				
Day surgery	276 (66.3)	169 (65.0)	107 (68.6)	0.453
Hospitalized	140 (33.6)	91 (35.0)	49 (31.4)	
Surgical margins				
Positive	63 (12.7)	31 (9.9)	32 (17.2)	0.019
Missing	1 (0.2)	1 (0.3)	0 (0.0)	

LVI, lymphatic/vascular invasion; PNI, perineural invasion.

**Table 2:** Univariate and multivariate regression analyses of study variables.

Study Variables	Univariate		Multivariate	
	OR (95%CI)	*p*-Value	OR (95%CI)	*p*-Value
Female	1.32 (0.83–2.11)	0.230		
Age, mean yrs ± SD (range)	1.01 (0.99–1.03)	0.128		
Number of lesion(s)/patient, *n* (%)				
1	ref.			
≥2	0.93 (0.52–1.63)	0.802		
Lesion diameter *n*, mean ± SD (range)	1.19 (1.05–1.36)	0.008		
Degree of differentiation				
Well/moderately differentiated	ref.			
Poorly differentiated/undifferentiated	1.93 (1.19–3.12)	0.007		
Tumor invasion				
In situ	ref.		ref.	
Invasive	7.03 (4.19–111.78)	<0.001	4.69 (2.55–8.16)	<0.001
Microinvasive	0.37 (0.17–0.84)	0.017	0.06 (0.01–0.29)	0.001
Depth of invasion				
Papillary dermis	ref.		ref.	
Reticular dermis	6.32 (2.91–13.73)	<0.001	9.27 (3.72–23.10)	<0.001
Dermis (unspecified)	10.49 (5.56–19.79)	<0.001	7.71 (3.87–15.32)	<0.001
Hypodermis	8.54 (4.32–16.89)	<0.001	7.27 (3.41–15.46)	<0.001
Muscle tissue	2.05 (0.72–5.85)	0.178	1.39 (0.42–4.56)	0.581
LVI and PNI				
LVI	-	-		
PNI	1.73 (0.86–3.48)	0.123		
Time to surgery, *n*, mean months ± SD (range)	0.92 (0.83–1.03)	0.158		
Surgical technique				
Direct closure	ref.		ref.	
Dermo-epidermal graft	2.57 (1.60–4.14)	<0.001	2.06 (1.09–3.88)	0.025
Dermal matrix	1.41 (0.80–2.51)	0.232	0.90 (0.40–2.00)	0.802
Flap	1.00 (0.60–1.69)	0.975	0.86 (0.44–1.71)	0.668
Hospitalization				
Day surgery	ref.			
Hospitalized	0.99 (0.66–1.47)	0.970		
Surgical margins				
Positive	1.87 (1.10–3.19)	0.020	3.21 (1.44–7.17)	0.004
Nodal metastases	3.38 (0.30–37.53)	0.321		

LVI, lymphatic/vascular invasion; PNI, perineural invasion.

## Data Availability

The data presented in this study are available in this article.
